# Supplementary material for: Chronic Plasma Exposure to Kinase Inhibitors in Patients with Oncogene-Addicted Non-Small Cell Lung Cancer
Source: Cancers (Basel). 2020 Dec 14;12(12):3758. doi: 10.3390/cancers12123758 (PMC7764991; doi:10.3390/cancers12123758)
Supplement: Supplementary file 1 [file cancers-12-03758-s001.pdf]

cancers

MDPI

Supplementary Materials: Chronic Plasma Exposure to Kinase Inhibitors in Patients with Oncogene-Addicted Non-Small Cell Lung Cancer

Arthur Geraud, Laura Mezquita, Edouard Auclin, David Combarel, Julia Delahousse, Paul Gougis, Christophe Massard, Cécile Jovelet, Caroline Caramella, Julien Adam, Charles Naltet, Pernelle Lavaud, Anas Gazzah, Ludovic Lacroix, Etienne Rouleau, Damien Vasseur, Olivier Mir, David Planchard, Angelo Paci and Benjamin Besse

Estimation of plasmatic residual concentration

Target drug monitoring (TDM) compromises the measurement and interpretation of drug concentrations in biological fluids to assist in the determination of drug dosage for the individual patient [1]. This strategy is used in clinical practice for therapeutic drug classes including antibiotics, immunosuppressors, antiepileptic and antiarrhythmic agents. Good candidates for TDM are those with a narrow therapeutic window and a direct relationship between plasma concentration and efficacy or toxicity (PK-PD relationship) [1].

The pharmacokinetic steady state is usually reached in two to three weeks and depends on the half-life of the drug, however data on the dynamic evolution of kinase inhibitors plasma concentrations are limited.

A retrospective study measured the plasma concentration of sorafenib every 15 days by liquid chromatography in a population of hepatocellular carcinoma patients, from initiation of treatment to progression [2] This showed that exposure after three months of treatment was lower than after one month of treatment. We thus defined chronic plasma exposure as a treatment duration of at least three months.

Plasma concentration was evaluated by the area under the curve (AUC) or residual plasma concentration, which is defined as the time just before the drug administration.

Precise measurement of trough concentrations is not easy in real-life due to the constraints of the logistics of a blood collection at a precise time and the fact that the timing of administration varies from patient to patient. According to the dosing time with respect to the last dose, we defined three situations: optimal, evaluable and non-interpretable.

- **Optimal**: the optimal concentration corresponds to the true residual plasma concentration at steady state, in the blood collection performed immediately before the next administration.

- **Evaluable**: the residual plasma concentration is estimated by an extrapolation method from known pharmacokinetic parameters (distribution volume, half-life, clearance) and from data obtained in population pharmacokinetic models[3]. This estimate of standard trough concentration (C_{min}, std) is only possible when blood samples were collected at steady state or during the terminal elimination of the drug phase since, in this phase, the elimination rate is linear [[3,4]].

- C (min, std) = C(t)* 0.5 ^ (Delta (t)/ t1/2)

- C (min, std) = C(t)* exp (k(e) x Delta (t))

Delta t = t - tau, tau is 24 hours when collecting a sample once a day, or 12 hours when collecting samples twice a day, k(e) is the elimination rate constant.

Pharmacokinetic parameters and PK-POP study are summarized in SupplementaryTable 2.

- Not interpretable: extrapolation not feasible for samples taken during the plasma peak period.

Somatic molecular analysis

ALK fusion was assessed by immunohistochemistry (IHC) or fluorescence in situ hybridization (FISH), while *ROS1* FISH was used to assessed fusions and RT-PCR or next generation sequencing (NGS) was used to assess mutations and/or other alterations.

Blood sample collections and ctDNA (for mutational analyses) were collected longitudinally, including at the time of disease radiological evaluation.

Plasma was isolated and ctDNA analysis was performed centrally (Gustave Roussy, France) using a targeted panel.

The panel used is described in Supplementary Table 4.

Panels used for blood samples (liquid biopsy):

- CHP2 (Ion AmpliSeq Cancer Hotspot Panel v2 (CHP2) designed to amplify 207 amplicons covering 50 genes (Thermo Fisher Scientific)).
- Oncomine lung (Oncomine Lung ctDNA Assay contains 35 amplicons covering 11 genes (Thermo Fisher Scientific). A unique molecular identifier is combined with each single DNA molecule.

Panels used for tissue samples:

- MOSC4 (Ion AmpliSeq MOSC4 designed to cover 82 genes, combining two other panels (CHP2 + Safir02).
- OCAV3 (Ion AmpliSeq Oncomine Comprehensive Assay V3 enables detection of mutations across 161 genes, genes fusion and copy number variations [Thermo Fisher Scientific]).
- SentosaSQ NSCLC (Sentosa SQ Non-Small Cell Lung Cancer panel targets 11 genes with 28 amplicons [Vela Diagnostics]).

Statistical analysis

Median values (interquartile range) and frequencies (percentage) were provided for descriptions of continuous and categorical variables, respectively.

Medians and proportions were compared using the Student's t-test and chi-square test (or Fisher's exact test, if appropriate), respectively.

Time to treatment failure was defined as the time between kinase inhibitors initiation and progression.

Overall survival (OS) was defined as the time between kinase inhibitors initiation and death from any cause. Time to treatment failure and OS were estimated using the Kaplan-Meier method and described using median values with their 95% confidence intervals (95% CI).

Follow-up was calculated using the reverse Kaplan-Meier method. Correlation between exposure and *T790M* mutation occurrence was evaluated with a Pearson correlation coefficient.

All statistical analyses were performed with R studio version 2.15.2, p-values & p-values < 0.05 were considered statistically significant and all tests were two-sided.

Supplementary material references

1. De Jonge, M.E.; Huitema, A.D.R.; Schellens, J.H.M.; Rodenhuis, S.; Beijnen, J.H. Individualised cancer chemotherapy: strategies and performance of prospective studies on therapeutic drug monitoring with dose adaptation: a review. *Clin. Pharmacokinet.* **2005**, *44*, 147–173, doi:10.2165/00003088-200544020-00002.
2. Arrondeau, J.; Mir, O.; Boudou-Rouquette, P.; Coriat, R.; Ropert, S.; Dumas, G.; Rodrigues, M.J.; Rousseau, B.; Blanchet, B.; Goldwasser, F. Sorafenib exposure decreases over time in patients with hepatocellular carcinoma. *Invest. New Drugs* **2012**, *30*, 2046–2049, doi:10.1007/s10637-011-9764-8.
3. Wang, Y.; Chia, Y.L.; Nedelman, J.; Schran, H.; Mahon, F.-X.; Molimard, M. A therapeutic drug monitoring algorithm for refining the imatinib trough level obtained at different sampling times. *Ther. Drug Monit.* **2009**, *31*, 579–584, doi:10.1097/FTD.0b013e3181b2c8cf.
4. Hiemke, C.; Bergemann, N.; Clement, H.W.; Conca, A.; Deckert, J.; Domschke, K.; Eckermann, G.; Egberts, K.; Gerlach, M.; Greiner, C.; et al. Consensus Guidelines for Therapeutic Drug Monitoring in Neuropsychopharmacology: Update 2017. *Pharmacopsychiatry* **2018**, *51*, 9–62, doi:10.1055/s-0043-116492.

Table S1. Overview of practical target drug monitoring recommendation for KIs in thoracic oncology.

Kinase inhibitor	PK-PD relationship for efficacy	Outcome parameters associated with TDM targets	Proposed target for TDM (ng/ml)	Reference
Crizotinib	Yes	C_{min} & increased PFS	$C_{min} \geq 235$	[1–3]
Gefitinib	Yes	C_{min} & increased OS<br>D8/D3 C_{min} ratio & increased PFS	$C_{min} \geq 200$	[1–6]
Erlotinib	Yes	C_{min} & improved response	$C_{min} \geq 500$	[1-3,7,8]
Osimertinib	Not found	No relation found	$C_{min} \geq 166$	[1,2,9]
Dabrafenib	Not found	No relation found	$C_{min} \geq 96.1$	[1,2,10]
Trametinib	Yes	Increased PFS	$C_{min} \geq 10.6$	[1,2,11]

Abbreviations: C_{min}, plasma trough level; D, day; MKI, Multi kinase inhibitor; PFS, progression-free survival; PK-PD relationship, pharmacokinetic-pharmacodynamic relationship; OS, overall survival; TDM: target drug monitoring.

Table S2. Pharmacokinetics parameters of the KIs evaluated.

Kinase inhibitor	Bioavailability	T_{max} (h)	Distribution volume (L)	Half-life (h)	Metabolic pathway	Hepatic elimination	Reference
Crizotinib	43%	4-6	1772	42	CYP3A4	63%	[12,13]
Gefitinib	59%	3-7	41	41	CYP1A1, 2D6, 3A4, 3A5	68%	[14]
Erlotinib	59%	4	232	36.2	CYP3A4, 3A5, 1A2 and 1A1 extrahepatic	90%	[15,16]
Osimertinib	70%	6	918	44	CYP3A4	68%	[9,17]
Dabrafenib	95%	2	46	8	CYP2C8, 3A4	73%	[10]
Trametinib	72%	1.5	127	127	Deacetylation alone	80%	[11,18]

CYP: cytochrome P450.

Table S3. Patient baseline characteristics.

Characteristic	All patients (n=41)
**Patient age, mean (range), years**	65 (28-89)
**Sex, No. (%)**	
Female	26 (63%)
Male	15 (37%)
**Performance status, No. (%)**	
ECOG ≤1	39 (95%)
ECOG ≥2	2 (5%)
**Current smoker, No. (%)**	
Yes	4 (12%)
No	36 (88%)
**Concomitant PPI, No. (%)**	
Yes	13 (32%)
No	28 (68%)
**Histologic type, No. (%)**	
Adenocarcinoma	36 (88%)
Squamous cell carcinoma	2 (5%)
Squamous adenocarcinoma	3 (7%)

**Molecular alteration, No. (%)**	
ALK rearranged	3 (7%)
$BRAF^{V600E}$ mutation	5 (12%)
EGFR exon 19 deletion	18 (44%)
EGFR mutation, exon 21 (L858R)	9 (23%)
EGFR mutation, exon 21 (L833F)	1 (2%)
ROS-1 rearranged	2 (5%)
MET mutation, exon 14	2 (5%)
No molecular alteration (ALK, BRAF, EGFR, KRAS, MET, ROS-1)	1 (2%)

ECOG, Eastern Cooperative Oncology Group; PPI, proton pump inhibitors,.

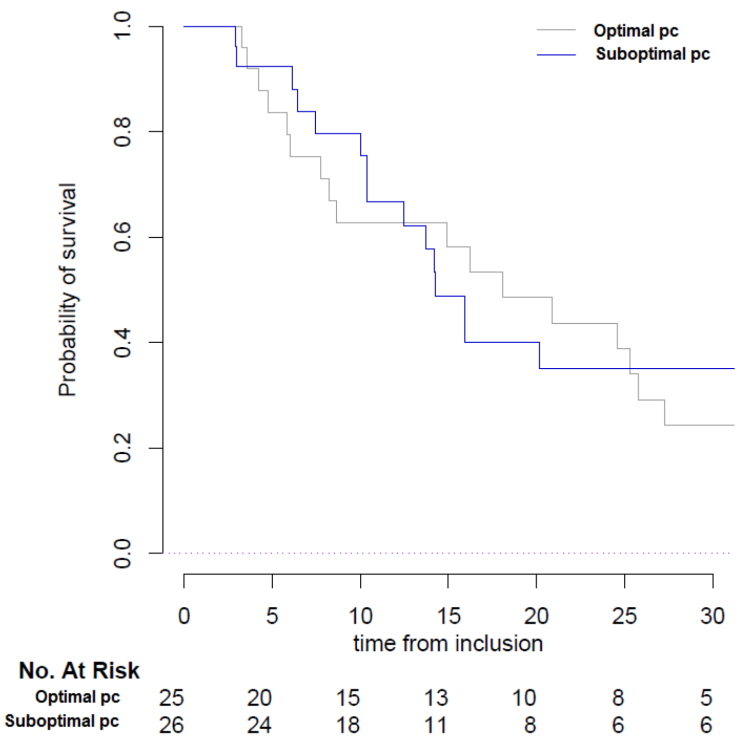

Suboptimal pc: Sub-optimal plasma concentration, Optimal pc: Sub-optimal plasma concentration

Figure S1. Kaplan-Meier curves of time to treatment failure according to plasma concentration.

References for Supplementary

1 Widmer N, Bardin C, Chatelut E, *et al.* Review of therapeutic drug monitoring of anticancer drugs part two--targeted therapies. *Eur J Cancer Oxf Engl 1990* 2014;**50**:2020–36. doi:10.1016/j.ejca.2014.04.015

2 Yu H, Steeghs N, Nijenhuis CM, *et al.* Practical guidelines for therapeutic drug monitoring of anticancer tyrosine kinase inhibitors: focus on the pharmacokinetic targets. *Clin Pharmacokinet* 2014;**53**:305–25. doi:10.1007/s40262-014-0137-2

3 Verheijen RB, Yu H, Schellens JHM, *et al.* Practical Recommendations for Therapeutic Drug Monitoring of Kinase Inhibitors in Oncology. *Clin Pharmacol Ther* 2017;**102**:765–76. doi:10.1002/cpt.787

4 Song Z, Zhang Y. Efficacy of gefitinib or erlotinib in patients with squamous cell lung cancer. *Arch Med Sci AMS* 2015;**11**:164–8. doi:10.5114/aoms.2013.39234

5 Nakamura Y, Sano K, Soda H, *et al.* Pharmacokinetics of gefitinib predicts antitumor activity for advanced non-small cell lung cancer. *J Thorac Oncol Off Publ Int Assoc Study Lung Cancer* 2010;**5**:1404–9. doi:10.1097/JTO.0b013e3181e59a7b

6 Zhao Y-Y, Li S, Zhang Y, *et al.* The relationship between drug exposure and clinical outcomes of non-small cell lung cancer patients treated with gefitinib. *Med Oncol Northwood Lond Engl* 2011;**28**:697–702. doi:10.1007/s12032-010-9541-0

7 Mir O, Blanchet B, Goldwasser F. Drug-induced effects on erlotinib metabolism. *N Engl J Med* 2011;**365**:379–80. doi:10.1056/NEJMc1105083

8 Soulieres D, Senzer NN, Vokes EE, *et al.* Multicenter phase II study of erlotinib, an oral epidermal growth factor receptor tyrosine kinase inhibitor, in patients with recurrent or metastatic squamous cell cancer of the head and neck. *J Clin Oncol Off J Am Soc Clin Oncol* 2004;**22**:77–85. doi:10.1200/JCO.2004.06.075

9 Brown K, Comisar C, Witjes H, *et al.* Population pharmacokinetics and exposure-response of osimertinib in patients with non-small cell lung cancer. *Br J Clin Pharmacol* 2017;**83**:1216–26. doi:10.1111/bcp.13223

10 Puszkiel A, Noé G, Bellesoeur A, *et al.* Clinical Pharmacokinetics and Pharmacodynamics of Dabrafenib. *Clin Pharmacokinet* 2019;**58**:451–67. doi:10.1007/s40262-018-0703-0

11 Ouellet D, Kassir N, Chiu J, *et al.* Population pharmacokinetics and exposure-response of trametinib, a MEK inhibitor, in patients with BRAF V600 mutation-positive melanoma. *Cancer Chemother Pharmacol* 2016;**77**:807–17. doi:10.1007/s00280-016-2993-y

12 Yamazaki S. Translational Pharmacokinetic-Pharmacodynamic Modeling from Nonclinical to Clinical Development: A Case Study of Anticancer Drug, Crizotinib. *AAPS J* 2012;**15**:354–66. doi:10.1208/s12248-012-9436-4

13 Hirota T, Muraki S, Ieiri I. Clinical Pharmacokinetics of Anaplastic Lymphoma Kinase Inhibitors in Non-Small-Cell Lung Cancer. *Clin Pharmacokinet* 2019;**58**:403–20. doi:10.1007/s40262-018-0689-7

14 White-Koning M, Civade E, Geoerger B, *et al.* Population analysis of erlotinib in adults and children reveals pharmacokinetic characteristics as the main factor explaining tolerance particularities in children. *Clin Cancer Res Off J Am Assoc Cancer Res* 2011;**17**:4862–71. doi:10.1158/1078-0432.CCR-10-3278

15 Lu J-F, Eppler SM, Wolf J, *et al.* Clinical pharmacokinetics of erlotinib in patients with solid tumors and exposure-safety relationship in patients with non-small cell lung cancer. *Clin Pharmacol Ther* 2006;**80**:136–45. doi:10.1016/j.clpt.2006.04.007

16 Thomas F, Rochaix P, White-Koning M, *et al.* Population pharmacokinetics of erlotinib and its pharmacokinetic/pharmacodynamic relationships in head and neck squamous cell carcinoma. *Eur J Cancer Oxf Engl 1990* 2009;**45**:2316–23. doi:10.1016/j.ejca.2009.05.007

17 Rossi A, Muscarella LA, Di Micco C, *et al.* Pharmacokinetic drug evaluation of osimertinib for the treatment of non-small cell lung cancer. *Expert Opin Drug Metab Toxicol* 2017;**13**:1281–8. doi:10.1080/17425255.2017.1401064

18 Cox DS, Papadopoulos K, Fang L, *et al.* Evaluation of the effects of food on the single-dose pharmacokinetics of trametinib, a first-in-class MEK inhibitor, in patients with cancer. *J Clin Pharmacol* 2013;**53**:946–54. doi:10.1002/jcph.115
